# Hydrothermal Synthesis of Lanthanide Stannates Pyrochlore Nanocrystals for Catalytic Combustion of Soot Particulates

**DOI:** 10.1155/2015/254165

**Published:** 2015-05-21

**Authors:** Xiaomin Zhang, Xuhui Liu, Peng Lu, Liguo Wang, Zhaoliang Zhang, Xiuju Wang, Zhongpeng Wang

**Affiliations:** School of Resources and Environment, University of Jinan, 336 Nanxinzhuangxi Road, Jinan 250022, China

## Abstract

Nanocrystalline La_2_Sn_2_O_7_ and La_2_Sn_1.8_Co_0.2_O_7_ with a phase-pure pyrochlore structure were synthesized by a hydrothermal method, and their catalytic activity was investigated for soot combustion. The as-synthesized catalysts presented relatively larger surface area, and pore volume, which was benefit to the gas molecule diffusion in the reaction. A uniform spherical structure with particle size of 200–500 nm was found in SEM. The samples via hydrothermal route are more active for catalytic soot combustion, ascribing to the spherical morphology, high surface area and improved oxygen mobility. After Co, the reducibility was improved and surface oxygen vacancy was produced, resulting in the enhanced activity and selectivity to CO_2_ formation.

## 1. Introduction

Soot particulates from diesel engine have caused serious problems to global environment and human health [1]. The automobile exhausts have been recognized as one of the important sources for the frequent haze weather in China now. Filtration and controllable regeneration within the exhaust stream are among the most promising methods for soot removal, while the key technology is oxidation catalysis [2, 3]. In a previous work, the kinetics of the soot-O_2_ reaction can be explained by the following reaction mechanism [4]:

(1) $$\begin{array}{c} {\text{O}}_{2}⟶2{\text{O}}_{\text{ad}}\; \end{array}$$

(2) $$\begin{array}{c} {\text{C}}_{\text{f}}+{\text{O}}_{\text{ad}}⟶{\text{C}}^{*}\left[\text{O}\right]\; \end{array}$$

(3) $$\begin{array}{c} {\text{C}}^{*}\left[\text{O}\right]+{\text{O}}_{\text{ad}}⟶\text{C}{\text{O}}_{2}\left(\text{g}\right)\left(+n{\text{C}}_{\text{f}}\right) \end{array}$$

(4) $$\begin{array}{c} {\text{C}}^{*}\left[\text{O}\right]+\frac{1}{2}{\text{O}}_{2}\left(\text{g}\right)⟶\text{C}{\text{O}}_{2}\left(\text{g}\right)\left(+n{\text{C}}_{\text{f}}\right) \end{array}$$

Gaseous O_2_ is adsorbed dissociatively on the catalyst surface (equation (1)), and the resulting atomic O_ad_ species then attack the reactive free carbon site, C_f_, to give an oxygen-containing active intermediate, C^*^[O] (equation (2)). The reaction between the C^*^[O] intermediate and either O_ad_ or gaseous O_2_ produces CO_2_ through reaction (3) or (4), reproducing the reactive C_f_ sites on soot surface.

In the past decades, many materials have been applied in oxidation catalysis, with transition-metal oxides [5–7], alkaline metal oxides [8–11], perovskite-like type oxides [12, 13], noble metals [3, 14], and ceria-based oxides [15, 16] being the outnumbering materials. However, these catalysts had some limitations in commercialization, such as the high cost of noble metals catalysts, poor stability of alkaline metal oxides, and weak activity in low temperature for transition-metal oxides. Thus, a cheap and efficient substitute with low-temperature activity and high selectivity is still desired.

During the past decade, pyrochlore-type oxides have emerged as important functional materials due to their interesting thermal, electrical, optical, magnetic, and catalytic properties [17]. Pyrochlores have the empirical formula A_2_B_2_O_7_, where A is a rare earth trivalent element and B is a tetravalent transition element. Their structure formula is often written as B_2_O_6_·A_2_O′, which emphasizes that the arrangement consists of a three-dimensional network of octahedron (B_2_O_6_) linked with an A_2_O′ tetrahedron in the interstices. For possibility of the existence of any pyrochlore compound, there are two criteria: (1) the ratio of the ionic radius of the cation at the A site to that at the B site must be between 1.46 and 1.80; (2) the chemical valences of various ions must make the compound to be charge neutrality [18]. The cations at the A site and the B site in the lattice can be replaced by the cations with different chemical valence or different oxidation reduction property to synthesize the various kinds of pyrochlore compounds with different physical or chemical properties, if the criteria can be satisfied. Substitutions of metals at the A or B sites in certain pyrochlore formulations can yield oxygen vacancies. As is known, the A site metal can be often replaced by alkali metal or other lanthanide elements whilst B site cation can be substituted by transition metals, such as Fe, Mn, Co, and Cu. By selecting A and B cations and/or introducing lattice defects, oxide pyrochlores exhibit improved redox property and catalytic activity. Among various pyrochlore oxides, lanthanide stannates, Ln_2_Sn_2_O_7_ (Ln=Y, La-Lu), have been well investigated in various environment-related high temperature catalytic reactions such as NO reduction [19], NO decomposition [20], CO oxidation [21], and catalytic combustion of methane [22, 23]. In our previous works [24], transition metal doped lanthanum stannate oxides with pyrochlore structure prepared by coprecipitation exhibited moderate catalytic activity for soot combustion in the presence of NO+O_2_.

With regard to the particle size and morphology, nanostructured materials are usually better for heterogeneous catalytic reactions due to their high surface area and quantum effects. Therefore, much emphasis has been laid on the shape control and the size control recently [15, 25, 26]. The surface particle sizes of nanometer material are small. Surface atoms on nanometer catalysts have extra and high surface energies and they are good at mobility; therefore, the contact between catalysts and soot can be improved even under loose contact conditions [27]. The development of adequate synthetic methods for the preparation of nanometric powders at low temperatures and atmospheric pressure is a task of great interest. Although various methods, such as conventional solid state approach [28], sol-gel processing, coprecipitation route [24], and combustion method [29], have been used to prepare La_2_Sn_2_O_7_, only hydrothermal route [30] has been proved to be a facial approach to fabricate La_2_Sn_2_O_7_ nanocrystallites with uniform particle sizes, high surface areas, regular shapes, and pure phases. It requires milder reaction conditions, compared with the high-temperature method.

Motivated by the above considerations, phase-pure lanthanum stannate pyrochlore oxides have been prepared via a simple hydrothermal technique. The catalysts showed nanosphere morphology, large surface area, and improved redox properties, which resulted in high catalytic activity and selectivity for soot combustion in the presence of oxygen.

## 2. Experimental

### 2.1. Catalyst Preparation

All the reagents were analytically pure, commercially available, and used without further purification. La_2_Sn_2_O_7_ and La_2_Sn_1.8_Co_0.2_O_7_ pyrochlore oxides were prepared with both hydrothermal method and coprecipitation method. The suitable amounts of La(NO_3_)_3_·6H_2_O, SnCl_4_·5H_2_O, Co(NO_3_)_2_·6H_2_O were dissolved in deionized water. The mixed salt solution (100 mL) and a basic solution (1 M NaOH) were simultaneously added dropwise into 100 mL of deionized water at constant pH (9.5 ± 0.5) under vigorous magnetic stirring. Precipitates were aged in suspension for 1 h under stirring in static air and then divided into two parts for different preparation methods. One part was transferred into a Teflon-lined stainless autoclave (200 mL capacity) that was filled with the mixture to 80% of the total volume. The autoclave was kept at a temperature 180°C for 18 h and then cooled to ambient temperature naturally. The resulting precipitates were washed with deionized water and ethanol thoroughly and dried at 80°C in an oven for 6 h before further characterization. The two pyrochlore oxides prepared by the hydrothermal method were denoted as LSO and CoLS. For comparison, the other part of precipitates was used for high temperature calcinations to prepare pyrochlore oxides. The precipitates was filtered and thoroughly washed with deionized water, then dried at 80°C for 12 h, and further dried at 120°C overnight. Finally, the two reference catalysts were obtained by calcination at 900°C for 5 h in air, denoted as LSO-c and CoLS-c, respectively.

### 2.2. Catalyst Characterization

XRD was carried out with a BRUKER-AXS D8 Advance X-ray diffractometer over a 2*θ* range from 10 to 80° at a scan rate of 3°/min. N_2_ adsorption-desorption isotherms were performed using a Micromeritics ASAP 2020 surface area analyzer after outgassing at 200°C for 5 h prior to analysis. The specific surface areas were calculated with the BET equation on the basis of the adsorption data. The morphology of the samples was examined with scanning electron microscopy (SEM, QUANTA FEG250). The particle size distribution histogram was measured with dynamic light scattering (DLS) using a Zetasizer Nano ZS instrument. Temperature-programmed reduction with H_2_ (H_2_-TPR) experiments was performed in a quartz reactor with a thermal conductivity detector (TCD) to monitor the H_2_ consumed. A 50 mg sample was pretreated* in situ* at 500°C for 0.5 h in a flow of O_2_ and cooled to room temperature in the presence of O_2_. TPR was conducted at 10°C/min up to 900°C in a 50 mL/min flow of 5 vol.% H_2_ in N_2_. The FTIR spectra were recorded on a Thermo Nicolet 6700 spectrometer over 400–4000 cm^−1^ after 32 scans at a resolution of 4 cm^−1^.

### 2.3. Catalytic Reactions

The model soot used in this study was Printex-U from Degussa with surface area of 93.5 m^2^/g. The mean agglomerate size measured using a laser particle size analyzer was about 177 nm [10].

The catalytic reactions for soot combustion were performed by a TPO technique in a fixed-bed flow reactor as described in our previous works [31, 32]. The soot was mixed with the catalyst in a weight ratio of 1 : 9 in an agate mortar for 10 min, which results in a tight contact. A 50 mg sample of the soot/catalyst mixture was pretreated in He (100 mL/min) at 200°C for 1 h to remove surface-adsorbed species. After cooling down to ambient temperature naturally and replacing the helium flow with the reaction gas flow (5% O_2_/He, flow rate 100 mL/min), the TPO was started at a heating rate of 4°C/min. The outlet gas was analyzed online by a gas chromatograph (GC) (SP-6890, Shandong Lunan Ruihong Chemical Instrument Corporation, China) with a flame ionization detector after separating them over a TDX-01 column and converting to methane over a Ni catalyst at 360°C. The sampling interval by the GC is 4 minutes. The characteristic temperatures from the TPO profiles, *T*_5_, *T*_50_, and *T*_90_, were defined as the temperatures at which 5%, 50%, and 90% of the soot is converted, respectively. The selectivity to CO_2_ formation (*S*_CO_2__) was defined as the percentage outlet CO_2_ concentration divided by the sum concentrations of the outlets CO_2_ and CO.

## 3. Results and Discussion

### 3.1. XRD Analysis

**Figure 1:**
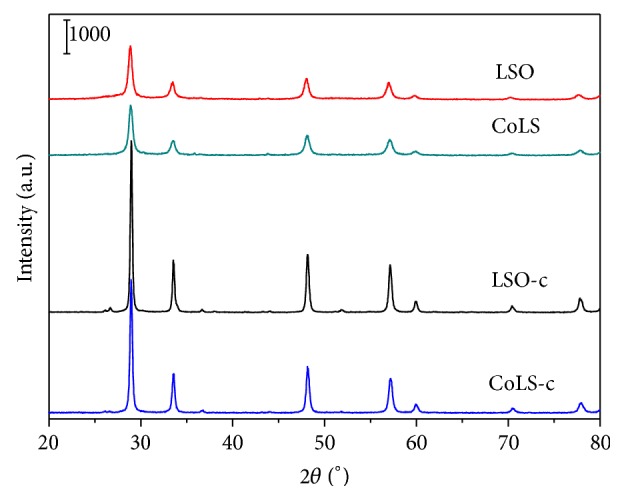
XRD patterns of pyrochlore oxide prepared by hydrothermal method and coprecipitation method.

The XRD patterns of the pyrochlore catalysts are shown in Figure 1. It can be seen that the four samples show typical diffraction patterns of La_2_Sn_2_O_7_ (JCPDS 13-0082) with pyrochlore structure conforming to the Fd-3m space group. Thus, single-phase lanthanum stannate pyrochlore oxides can be prepared by both hydrothermal and coprecipitation method. As expected, the samples via hydrothermal technique (LSO and CoLS) exhibit broad and weak diffraction peaks in comparison with those by coprecipitation method (LSO-c and CoLS-c). The crystallite size (*X*_*s*_) of the solids, listed in Table 1, was calculated from the (222) diffraction peak around 2*θ* value of 29° by using Debye-Scherrer equation. According to the equation, the crystallite size is in inverse proportion to the FWHM values of the peak [33]. The hydrothermal catalysts have a pyrochlore crystalline size of 19-20 nm, while the coprecipitation method produces samples with larger size of 30–40 nm. In addition, the characteristic diffractions of CoLS indicate that the Co cations are well distributed in the pyrochlore structure and a small quantity of Co in the pyrochlores does not change the crystal purity since there is no peak related with other compounds. Meanwhile, oxygen vacancies can be yielded with replacement of Sn^4+^ by a lower valence transition metal ion to maintain electrostatic charge neutrality. In this sense, the oxides should be expressed by La_2_Sn_1.8_Co_0.2_O_7−*δ*_ (*δ* = oxygen vacancy). The formed oxygen vacancies may influence the oxygen mobility in the oxidation reactions.

### 3.2. SEM Morphology

**Figure 2:**
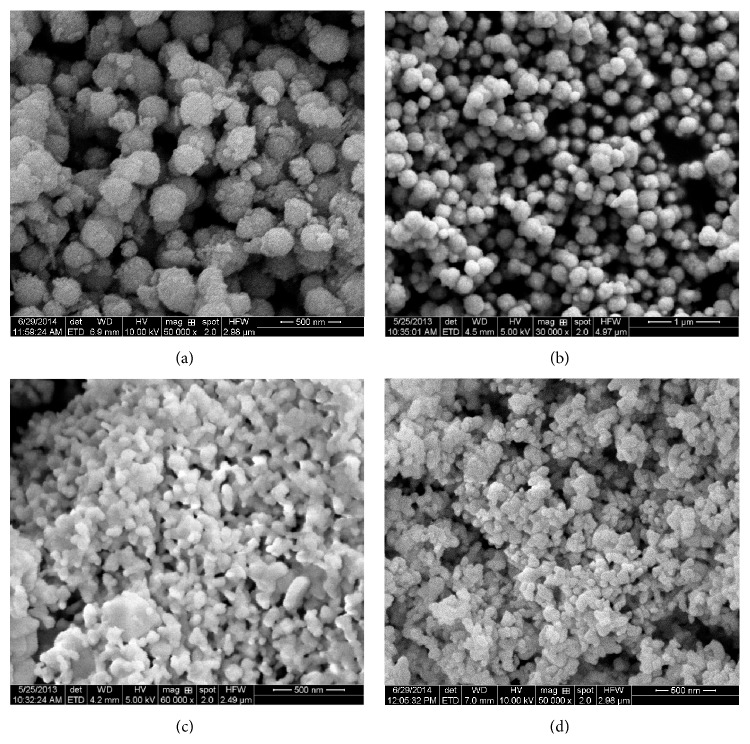
SEM micrographs of LSO (a), CoLS (b), LSO-c (c), and CoLS-c (d) samples.

**Figure 3:**
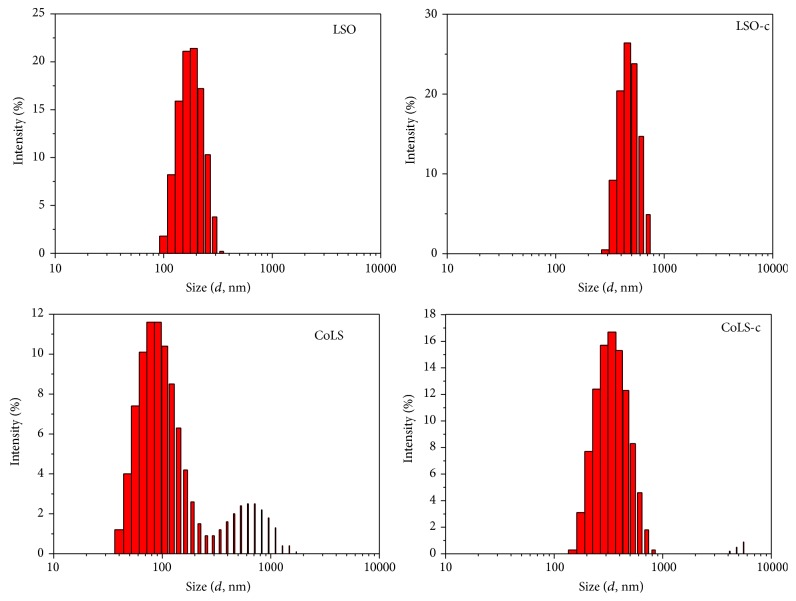
Histograms of particle size distribution.

The SEM morphology of the samples is shown in Figure 2. SEM observation revealed that the hydrothermal catalysts (Figures 2(a) and 2(b)) show nearly spherical particle with rough surfaces and particle size of 200–500 nm, while coprecipitation samples (Figures 2(c) and 2(d)) demonstrate aggregation of particles. More uniform and better dispersed particles were found for CoLS sample. The spherical structure of the catalysts can increase the contact area between soot and catalyst and facilitate oxygen migration in the catalytic reactions, thus improving the catalytic activity for soot combustion. SEMs did not show any significant difference between the morphological features of the as-synthesized LSO-c and CoLS-c samples. The aggregation of the LSO-c and CoLS-c particles may be caused by the high calcination temperature and the high surface energy [34].  Figure 3 showed a narrow particle distribution centered at 100–500 nm. In this case, most of histograms are unimodal except for CoLS sample. According to the histogram of particle size distribution, the mean diameter of particles coincides with the results from SEM images.

### 3.3. N_2_ Adsorption-Desorption Characterization

N_2_ physisorption experiments were carried out to examine the texture characteristics of the samples. All the oxides samples presented adsorption isotherms of type IV in the IUPAC classification, which are representative of mesoporous materials with no or few micropores and strong interaction between adsorbent and adsorbate molecules. The hysteresis loops at a high relative pressure indicate a capillary condensation of adsorbate in the meso- and macropores of the solids. The pyrochlore oxides resemble H3 type hysteresis loops, which is usually given by adsorbents with slit-shaped pores.

**Figure 4:**
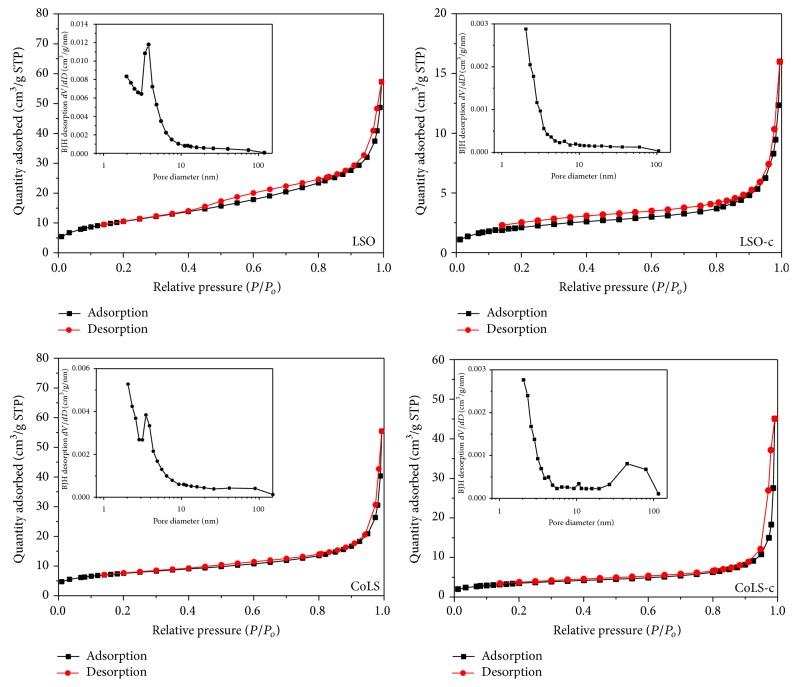
N_2_ adsorption-desorption isotherms and pore size distribution of the samples.

As calculated from the pore size distribution curves (Figure 4 inset), most of the pores fall in mesosize range (2 nm < *r*_*p*_ < 50 nm). The BET specific surface area (*S*_BET_), total pore volume (*V*_*P*_), and average pore diameter (*D*_*P*_) of the mixed oxides determined from isotherms are listed in Table 1. The catalysts via hydrothermal method show larger surface areas and pore volumes, which may be related to their smaller crystallite sizes. The lower surface area of coprecipitation samples may be due to the aggregation of the particles as can be seen from the SEM morphology. The LSO sample exhibits high surface area of 39.0 m^2^/g and large pore volume of 0.057 cm^3^/g.

### 3.4. FTIR Characterization

**Figure 5:**
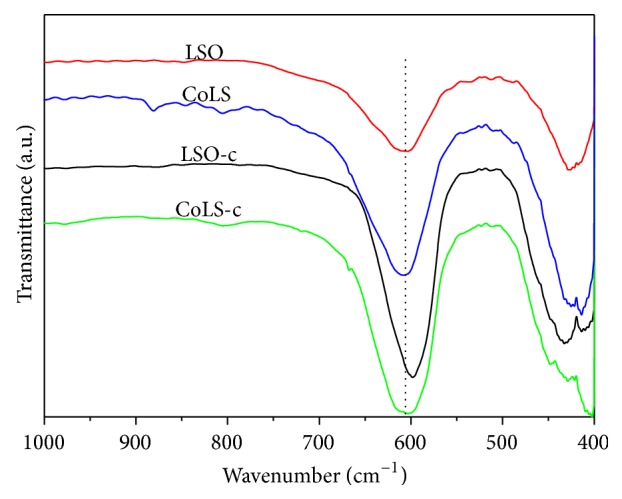
Infrared spectra of the Sn-O stretching vibration of samples.

In pyrochlores the metal ions are situated in two different sublattices designated as tetrahedral (A site) and octahedral (B site) sites according to the geometrical configuration of the neighboring oxygen [35]. It has been reported that pyrochlore oxides exhibit seven IR bands in the range of 750–50 cm^−1^ originating from vibration and bending of metal-oxygen bonds [23]. The band at about 600 cm^−1^ is from the B-O stretching vibrations in the BO_6_ octahedron and the band around 400 cm^−1^ to the A-O stretching vibrations. The IR spectra of the pyrochlore catalysts recorded in the range of 400–1000 cm^−1^ are shown in Figure 5. At about 600 cm^−1^, one broad band ascribable to Sn-O stretching vibration was observed for each sample. The *ν*(Sn-O) based on IR spectra of the catalysts is shown in Table 1. The *ν*(Sn-O) of hydrothermal samples shifts to high frequency compared to the coprecipitation samples, indicating changes in Sn-O bond strength. The differences in *ν*(Sn-O) resulting from different preparation conditions were also reported [36]. These changes in Sn-O bond strength may influence oxygen mobility of the catalysts because the Sn-O bond is responsible for the release of lattice oxygen when enough energy is provided [24]. This can also be related to the TPR results where the reducibility of the samples was enhanced after Co doped in the La_2_Sn_2_O_7_ pyrochlore.

### 3.5. Temperature-Programmed Reduction with H_2_

**Figure 6:**
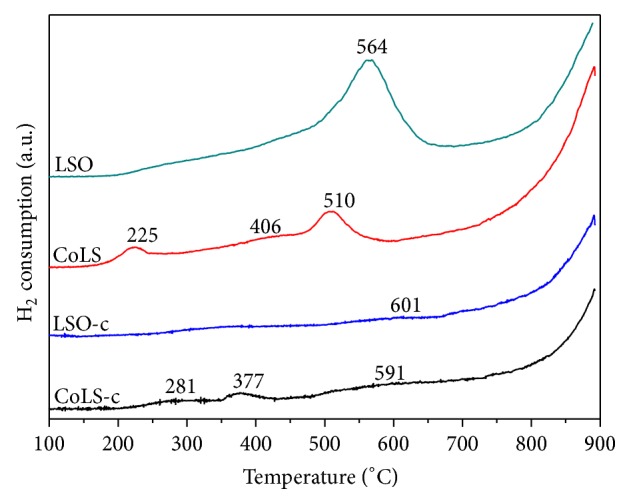
H_2_-TPR profiles of pyrochlore samples.

The H_2_-TPR profiles of pyrochlores oxides, presented in Figure 6, show that the materials possess considerably different reducibility. LSO sample displays a wide low temperature peak at 564°C and a high temperature peak above 700°C, which belongs to the reduction of Sn^4+^ to Sn^2+^ and Sn^2+^ to Sn^0^, respectively. A further reduction behavior is observed on the catalyst of CoLS, which presents a large H_2_ consumption peak around 225°C attributed to the reduction of Co^3+^ to Co^2+^. The complete reduction of Co^2+^ to Co^0^ only takes place at higher temperature (about 406°C). Similar reduction phenomena were observed on the LSO-c and CoLS-c samples. In comparison with the samples by coprecipitation method, the catalysts via hydrothermal route show enhanced reduction of Sn^4+^ to Sn^2+^, which may be linked to the changes in Sn-O bond strength. Furthermore, small amounts of Sn replaced by cobalt metals influence the reduction behavior, especially on the low temperature range, which is due to the interactions between Sn and cobalt metals. It can be seen from the onset temperature for reduction that the reducibility decreases by the following order: CoLS>LSO>CoLS-c>LSO-c.

### 3.6. Catalytic Performance of Soot Oxidation

**Figure 7:**
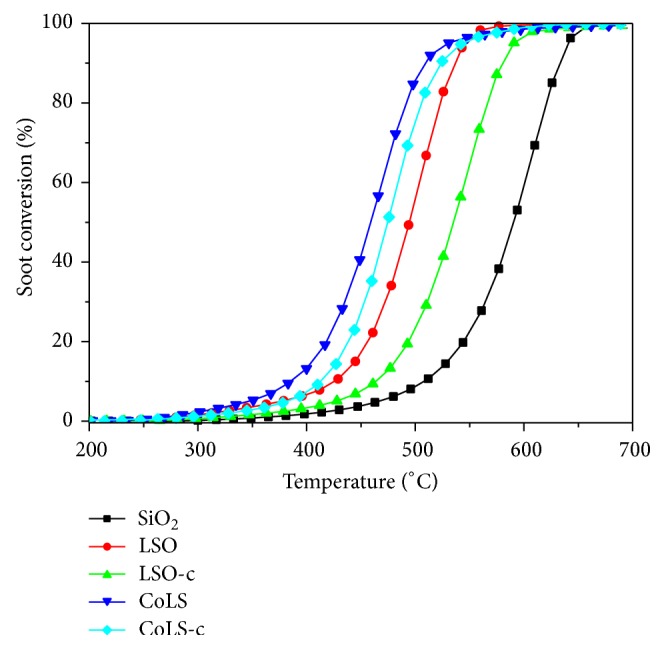
Temperature dependence of soot catalytic combustion over pyrochlore catalysts.

The soot conversion profiles obtained during catalytic tests with O_2_ are plotted as a function of temperature in Figure 7. The derived parameters of *T*_5_, *T*_50_, *T*_90_, and *S*_CO_2__ are listed in Table 2. As shown in Figure 7, the blank experiment with O_2_ was performed mixing the soot with SiO_2_, and the ignition temperature (*T*_5_) was 470°C. In comparison with the noncatalyzed soot oxidation, soot conversion curves over the pyrochlore catalysts shift to lower temperature range with *T*_5_, *T*_50_, and *T*_90_ decreased and *S*_CO_2__ increased. As can be seen from Figure 7 and Table 2, the samples by hydrothermal method are more active than that by coprecipitation method, which can be ascribed to the following reasons: (1) the spherical morphology and better dispersed particles with smaller size can increase the contact points with soot; (2) high surface area and pore volumes will favor of gas molecule diffusion in the pores; (3) the improved oxygen mobility induced from the changes in Sn-O bond strength.

Furthermore, the Co-doped pyrochlores exhibit higher activity and selectivity than the undoped catalysts, which may be ascribed to the enhancement of reducibility confirmed by TPR. Among all samples, CoLS possessed the best activity with soot ignition temperature of 342°C and high selectivity to CO_2_ formation of 96.9%. It is well known that the incorporation of functional dopant to A or B sites in the pyrochlore lattice leads to defect structures and thus creates more active sites to make catalytic activity improved [23]. Reactivity of metal oxides is largely dependent on the degree of the coordinative unsaturation of the metal ions. The incorporation of cobalt ions in the pyrochlore structure leads to the formation of oxygen vacancies and makes local structure changed where the coordinative unsaturation of specific Sn ions increases. In general, the high activity of the CoLS catalyst is attributed to the enhancement of reducibility and the surface oxygen vacancy formed during the breakage of metal-oxygen lattice bond besides the three points mentioned above.

## 4. Conclusions

Nanocrystalline La_2_Sn_2_O_7_ and La_2_Sn_1.8_Co_0.2_O_7_ pyrochlore catalysts were synthesized by hydrothermal method at temperatures as low as 180°C. Relatively uniform spherical structure with particle size of 200–500 nm was produced via hydrothermal route. These catalysts also presented larger specific surface area and pore volume, which were benefit to the adsorption and desorption of gas molecules in the catalytic reaction. Both oxygen mobility and reducibility of the catalysts were improved which may be linked to the changes in Sn-O bond strength. The samples via hydrothermal route are more active for catalytic soot combustion than that by coprecipitation method, which may be related to the spherical morphology, high surface area, and improved oxygen mobility. Besides the three points mentioned above, the Co-doped pyrochlores exhibit higher activities which may be ascribed to the enhancement of reducibility and the formed surface oxygen vacancy. Among all samples, La_2_Sn_1.8_Co_0.2_O_7_ possessed the best activity with soot ignition temperature of 342°C and high selectivity to CO_2_ formation of 96.9% under O_2_ atmosphere.

## Figures and Tables

**Table 1:** Structural information of the pyrochlore catalysts.

Samples	*S*_BET_ (m^2^/g)	*V*_*P*_ (cm^3^/g)	*D*_*P*_ (nm)	*ν*(Sn-O) (cm^−1^)	*X*_*s*_ (nm)
LSO	39.0	0.057	5.9	605	20.3
LSO-c	7.6	0.012	6.7	598	40.7
CoLS	27.0	0.041	6.0	607	19.3
CoLS-c	12.7	0.023	7.3	603	32.4

*S*_BET_: the BET specific surface areas; *V*_*P*_: total pore volume; *D*_*P*_: average pore diameter; *X*_*s*_: crystallite size.

**Table 2:** The catalytic performance of catalysts for soot combustion.

Samples	*T*_5_ (°C)	*T*_50_ (°C)	*T*_90_ (°C)	*S*_CO_2__ (%)
SiO_2_	470	590	633	37.8
LSO	377	495	535	95.4
LSO-c	426	534	580	79.7
CoLS	342	457	507	96.9
CoLS-c	383	473	524	97.7
